# Combined, sequential procedure for determination of ^137^Cs, ^40^K, ^63^Ni, ^90^Sr, ^230,232^Th, ^234,238^U, ^237^Np, ^238,239+240^Pu and ^241^Am applied for study on contamination of soils near Żarnowiec Lake (northern Poland)

**DOI:** 10.1007/s10967-016-4835-0

**Published:** 2016-04-28

**Authors:** Jerzy W. Mietelski, Renata Kierepko, Edyta Łokas, Anna Cwanek, Krzysztof Kleszcz, Ewa Tomankiewicz, Tomasz Mróz, Robert Anczkiewicz, Mirosław Szałkowski, Bogdan Wąs, Mirosław Bartyzel, Ryszard Misiak

**Affiliations:** 1 Institute of Nuclear Physics, Polish Academy of Sciences, Krakow, Poland; 2 State Higher Vocational School in Tarnow, Tarnow, Poland; 3 Pedagogical University of Krakow, Krakow, Poland; 4 Institute of Geological Sciences (ING), Polish Academy of Sciences, Krakow, Poland

**Keywords:** Radiochemical sequential procedure, Environmental radioactivity, Actinides in soil, Plutonium, ^237^Np, ^63^Ni, Polish nuclear power plant to be

## Abstract

The paper summarizes results of investigation of the current state of radioactive contamination on site being under consideration for planned nuclear power plant in northern Poland. Thanks to use of sequential procedure it was possible to determine activity concentrations for radioisotopes of nine elements, both natural and artificial. Results show that observed levels of radioactive contamination are rather typical for central Europe and global fallout is dominant factor of presence of artificial radionuclides. The total deposition for artificial radionuclides revealed maxima equal to 1747 ± 121 Bq/m^2^ for ^137^Cs, 3854 ± 158 Bq/m^2^ for ^90^Sr, 101 ± 23 mBq/m^2^ for ^237^Np, 57.7 ± 6.0 Bq/m^2^ for ^241^Am, 3.27 ± 0.80 Bq/m^2^ for ^238^Pu and 68.5 ± 5.0 Bq/m^2^ for ^239+240^Pu.

## Introduction

Poland is approaching to join the countries possessing nuclear power plants (NPPs). This opens new challenges to environmental radioactivity studies. Present state of environmental monitoring in Poland is mostly affected by lesson learnt from Chernobyl accident, where the most important radionuclides in the fallout were caesium isotopes: ^137^Cs and ^134^Cs. This was the reason for choosing high resolution, semiconductor gamma spectrometry as main technique used in environmental radioactivity laboratories of Poland. However, the environmental impact of nuclear power plant has to be monitored much wider. Releases of tritium and isotopes of noble gasses are the most possible events, but their detection on environmental levels is very limited in our country. Some possible scenarios of accidental releases of radionuclides from a reactor or from stored spent fuel as well as different environmental behaviour of caesium and some other radionuclides require development of measurements techniques for wide palette of long lived radionuclides. Although caesium can be released together with other nuclides, it can be fractionated and accumulated in different parts of environment.

For many years in our laboratory we have been developing sequential measurement procedure, which allows us to measure activity concentrations of many radionuclides in a single environmental sample [1, 2]. There are many motivations for such measurement policy. Environmental samples are sometimes quite unique—the mass of the sample collected frequently on remote locations is limited due to the need of hand transportation. The advantage of analysing as many radionuclides as possible is obvious [3, 4]. The information obtained from the ratios between different radionuclides significantly enlarges our knowledge on the sample, pointing to the source (in case of fresh fallout) or providing the data on the radionuclides fractionation. Moreover, dissolution of the sample often requires massive efforts and man power. All this supports concept of developing a single, sequential radiochemical procedure, which applied to a single environmental sample, after single mineralization, can give information on many radionuclides. Unfortunately, it happens that using the sequential method results in lowering radiochemical recovery for one or more elements. Recently in our laboratory sub-procedures for ^63^Ni and ^237^Np were included into existing sequential procedure, which starts from gamma spectrometric measurements for gamma emitters, followed by radiochemical analyses of ^241^Am, Pu, Th, ^237^Np, U and ^90^Sr. The whole procedure is designed to suit different types of environmental samples including soils, sediments, peat, etc.

The study was related to Polish project for nuclear energy development entitled “Technologies supporting the development of safe nuclear power”. We intended to demonstrate capability of our procedure to investigate the “time zero state” of radioactive contamination on site of planned nuclear power plant, where the “time zero state” is the state before any nuclear power plant activity in considered area. Since the decision on the exact location is still being postponed to the future, our study was conducted on Żarnowiec Lake area, which is one of possible options. The knowledge about radioactive pollution of this region of northern Poland is limited, especially this work is the first Polish one, which gives data on ^237^Np activity concentration and deposition in soil profiles.

## Materials and methods

### Sampling

**Fig. 1:**
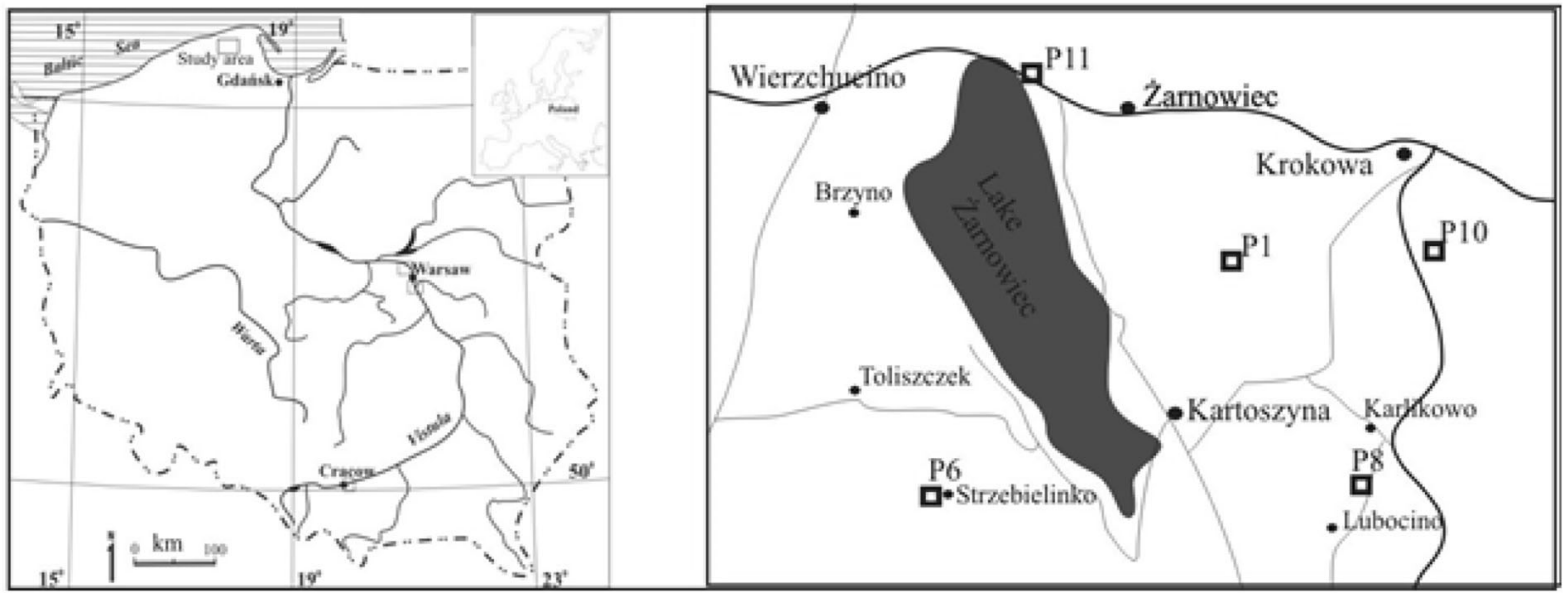
Location of sampling sites in map of Poland (*left*) and in details (*right*)

The investigated area is located in north part of Poland around the Lake Żarnowiec. Figure 1 presents the map of studied area and sampling locations. Sampling was done at the end of April 2014. Five cores of 12.5–24.5 cm length were collected using 10 cm diameter PCV cylinder pushed into the soil. The cross-section of sampling cylinder was 79 cm^2^ and this value was used in deposition calculations. The inventory of the profile was calculated according to the following equation:

1 $$D = \sum\limits_{i} {C_{i} \cdot \frac{{m_{i} }}{S}}$$

where:

*D—*inventory (total deposition) of given profile.

*C*_*i*_*—*activity concentration of soil layer of given profile.

*m*_*i*_*—*dry mass of whole soil layer of given profile.

*S—*cross-sectional area.

This investigated area lies near to constructed in the past, but abandoned and never finished Nuclear Power Plant, which was supposed to be the first one in Poland. Sample codes were P1, P6, P8, P10, P11; those were numbers attributed to wider study (the governmental research project), where dose rate was also investigated in many more locations. For consistence we keep those codes here.

### Gamma spectrometric measurements

All soil profiles were cut into layers of 2–3 cm height. Separated slices were dried at 105 °C, homogenized, and sub-samples of 140 ml were taken for gamma spectrometric measurement. We used spectrometer made by Silena SPA (Italy) with HPGe coaxial detector with resolution of 2.0 keV (at 1173 keV) shielded with 10 cm of lead, 3 mm of cadmium and 2 cm of copper inner lining. Spectrometer was calibrated using Mix Gamma source CBSS-2 (nr 141111-1329021) produced by Inspectorate for Ionizing Radiation, Prague (Czech Republic), in form of the same 140 ml vessel as used for the samples.

### Radiochemical procedure

After gamma spectrometric measurements some layers of the profile were combined into sets following their morphology (colour, structure etc.) and results on gamma spectrometric measurements. Sample codes are P1a, b, c, P6a, b, c, P8a, b, c, P10a, b, c and P11a, b, c. Resulting combined sets contained slices from 2.5 to 11.5 cm height and after careful mixing were subject of further radiochemical analysis. Each sub-samples (~10 g) were ashed in 400 °C for about 5 h. Then, radioactive tracers (^242^Pu, ^232^U, ^229^Th, ^239^Np, ^243^Am, ^85^Sr) and stable nickel (as a tracer for ^63^Ni analyses) were added, all determined by weight using analytical balance. ^239^Np as an isotope with short half live time (2.36 d) [5] was milked from ^243^Am generator and spiked to the samples directly before digestion [6]. The same activity of ^239^Np that was added to the samples was also kept for comparative measurement in order to estimate radiochemical recovery of the used method (by gamma spectrometry) and to check purity of obtained tracer (by mass spectrometry). This approach allows to reduce uncertainties.

**Fig. 2:**
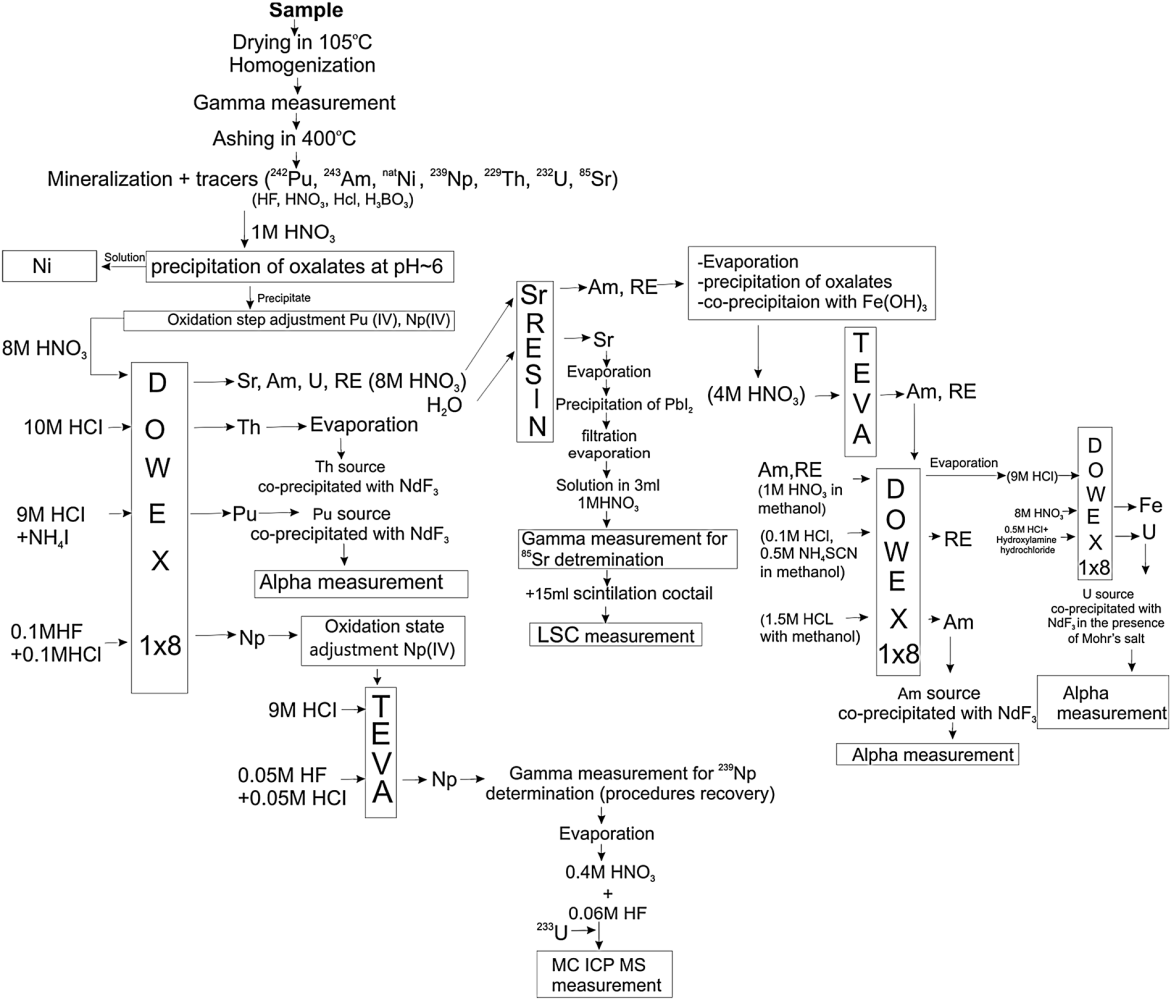
A scheme of sequential radiochemical procedure applied in present work

The radiochemical procedure (Fig. 2) started with complete wet digestion using concentrated HF, HNO_3_, HCl and small addition of H_3_BO_3_. Finally, sample solution was converted to 1 M HNO_3_.

At first, nickel was separated. For this purpose, the pH of previously obtained solution was adjusted to 6 using aqueous ammonia. It resulted in formation of precipitate containing all actinides and ^90^Sr whereas Ni stayed in the solution. In order to purify nickel fraction it was gently evaporated to dryness and converted to 6 M HCl. Then, the solution was passed through the pre-conditioned Dowex 2 × 8 column so that the residual traces of iron could be removed. After this purification, the solution was evaporated almost to dryness and slightly diluted with water. A few ml of 0.2 M ammonium citrate solution were added and the solution was adjusted to pH 8–9 using ammonium hydroxide. The whole mixture was loaded onto the pre-conditioned Ni-resin column (Triskem) and Ni was captured in red complex with dimethylglyoxime (DMG) [7]. Nickel was then rinsed with 3 M HNO_3_ and this final fraction was evaporated almost to dryness. After slight dilution with water it was transferred to LSC vial and was ready for activity measurement [8].

The precipitate was dissolved in hot concentrate nitric acid and back diluted to 1 M HNO_3_. The aim of next steps was adjustment appropriate oxidation states of Pu and Np. Firstly hydrazine (80 %) was added and the solution was heated for about 20 min. Secondly, after cooling the mixture to room temperature, 65 % HNO_3_ was added to produce a 8 M nitric acid solution [9]. It resulted in conversion oxidations states of plutonium and neptunium to Pu(IV) and Np(IV), respectively.

Subsequent separation of isotopes took place on column filled with anion exchange resin—Dowex 1 × 8 (Sigma-Aldrich). During passing sample solution through the column, there is no retention for uranium, americium and strontium, in contrast to thorium, plutonium and neptunium, which remains in the column. Th was eluted by 10 M HCl, Pu by 0.1 M NH_4_I in 9 M HCl and at the end Np by 0.1 M HCl + 0.1 M HF [10, 11]. The Pu fraction was evaporated and 65 % HNO_3_ was added and heated to remove iodine. Finally this fraction was dissolved in 20 ml of 1 M HCl. The TEVA (Triskem) resin was used for repurification of Np fraction from traces of Th and U. Before, it was necessary to convert the Np fraction in 5 M HNO_3_ solution. And again, after passing the feed solution through resin, Np was removed by mixture of 0.1 M HCl + 0.1 M HF. Next, Np fraction was converted to 5 M HNO_3_ and TEVA (Triskem) resin was used for repurification this fraction from traces of U and Th. Np was eluted by mixture of 0.05 M HCl and 0.05 M HF (Fig. 2).

^90^Sr was extracted directly from the Dowex 1 × 8 resin effluents (8 M HNO_3_) using column filled with Sr-Resin (Eichrom). Sr + Pb fraction was obtained by washing the column with deionised water. Subsequently, there was applied lead iodide precipitation in order to final purification from traces of ^210^Pb [12].

The Sr-resin effluent, containing Am, U and RE, was evaporated to small volume and diluted with deionized water. In order to dispose of matrix components, oxalic acid was added; the pH was adjusted to about 3 and the resultant CaC_2_O_4_ precipitate (with Am and U) was allowed to settle out. The precipitate was collected by centrifugation, and was re-dissolved in hot 65 % HNO_3_ and heated thereby decomposing the oxalate. Next, it was necessary to remove the Ca^2+^ (aq) matrix, so co-precipitation with Fe(OH)_3_ at pH ~ 9 was made. The precipitate (containing Am and U) was re-dissolved in 4 M HNO_3_ and passed through the TEVA (Triskem) resin; it was allowed to repurificate Am fraction from traces of Th. Finally, americium, uranium and rare earth (RE) components were removed using a conventional methanol-acid standard procedure on Dowex 1 × 8 resin [1–3].

Because of U fraction contained large amounts of iron present in the sample, it was necessary to dispose of. Thus this fraction was converted to 9 M HCl and passed through Dowex 1 × 8 resin. The iron was eluted by 8 M HNO_3_, while the uranium was removed by mixture of 6 ml of 0.5 M HCl with ca. 0.5 g of hydroxylamine hydrochloride.

### Measurements of separated fractions

Activity concentrations for plutonium, americium, thorium and uranium isotopes were determined using alpha spectrometers (Silena AlphaQuattro, Ortec Alpha Duo or Canberra 7401; all equipped with Canberra or Ortec ion-implanted silicon detectors). All alpha sources were prepared using NdF_3_ micro-coprecipitation method [13]. Activity concentrations for nickel and strontium isotopes were determined using 1414-003 Wallac Guardian liquid scintillation spectrometer. Samples were prepared in plastic scintillation vials (20 ml, by Triskem) in mixture of 5 ml of 0.5 M HNO_3_ with 15 ml of Gold Star LT2 scintillation cocktail. The same gamma spectrometer as described above was applied for determination of recovery of ^239^Np (99.6, 103.8, 106.1 keV) and ^85^Sr (514.0 keV) [5]. Recovery of nickel was determined using UV–Vis Helios Alpha spectrometer (Unicam Instruments). The method was based on the complex of nickel with alpha-furildioxime [8].

In order of neptunium concentration estimation, solution with Np isotopes (after gamma spectrometry measurement) was converted to 0.4 M HNO_3_ and 0.06 M HF solution and finally spiked by ^233^U for independent monitoring of the mass spectrometry measurements [10] based on the peak jumping method with secondary electron multiplier (SEM) ion counter. These measurements were carried out using Thermo Finningan Neptune multi collector, double sector mass spectrometer (MC-ICP-MS) installed at the Institute of Geological Sciences Polish Academy of Sciences.

### Quality assurance

Analysis quality was evaluated through the determination of radionuclides activity concentrations of IAEA reference materials (IAEA-375 and IAEA-385). A comparison of our obtained values for these materials with certified values is presented in Table 1. The results agreed well with recommended values and did not indicate any significant biases in analytical procedure. Blank samples made of reagents were also analysed. Average results for blanks were used for background corrections. Procedure was positively tested using reference materials spiked with NiCl_2_ as the tracers for radiochemical recovery. Since there are no reference materials with certified values for ^63^Ni available on the market, no other validation for ^63^Ni was possible.

**Table 1:** Quality control data: our results compared with certified values

Isotope/RM	Our result (Bq/kg)	Reference value (Bq/kg)	Recovery (%)
IAEA-375 soil			
^137^Cs	5066 ± 48^a^	5280 (5200–5260)	–
^238^Pu	0.09 ± 0.02	0.071 (0.056–0.085)	76
^239+240^Pu	0.34 ± 0.04	0.30 (0.26–0.34)	76
^241^Am	0.22 ± 0.03	0.13 (0.11–0.15)^c^	52
^234^U	20.5 ± 1.8	25 (17–32)	7
^238^U	19.5 ± 2.0	24.4 (19.0–29.8)	7
^232^Th	20.6 ± 1.4	20.5 (19.2–21.9)	42
^230^Th		nd	
^90^Sr	95 ± 6^a^	108 (101–114)	69
Ni		nd	
IAEA-385 sediment			
^137^Cs	36 ± 4^b^	33 (32.7–33.6)	–
^238^Pu	0.42 ± 0.06	0.44 (0.42–0.48)	79
^239+240^Pu	2.96 ± 0.30	2.96 (2.89–3.00)	79
^241^Am	3.37 ± 0.22	3.84 (3.78–4.01)	45
^234^U	27.9 ± 1.9	27 (26–28)	14
^238^U	29.4 ± 2.2	29 (28–30)	14
^232^Th	35.0 ± 2.6	33.7 (32.8–33.9)	22
^230^Th	32.9 ± 2.3	30.6 (30.0–33.6)	22
^90^Sr		0.58 (0.42–0.68)	
Ni		nd	

Uncertainties are 1 σ counting statistics

*nd* No data

^a^Corrected for decay to the reference date of IAEA soil 375 (31st December 1991)

^b^Corrected for decay to the reference date of IAEA sediment 385 (1st January 1996)

^c^Valid only for reference date of IAEA soil 375 due to unknown amount of ^241^Pu

Unfortunately, there is no reference material (soil) with well know activity concentration of ^237^Np, therefore quality assurance for this isotope was preserved and controlled by internal tracers ^239^Np (for digestion and separation part of procedures) and ^233^U (for mass spectrometry measurements).

To avoid any cross contamination, all PTFE and glass beakers were cleaned by washing them in vapours of boiling concentrated nitric acid for at least 1 h. Uncertainties are given with coverage factor *k* = 1. Our laboratory has Polish state accreditation (ISO 17025) for gamma spectrometric measurements and Pu alpha spectrometric analyses.

Table 2 presents results of recoveries. In most cases recoveries were satisfying with mean values close to 50 %. Some further improvements of the method seem to be required in case of nickel, which has the lowest values of recoveries (mean value close to 8 %, ranging from 30 to 3 %). The uranium recoveries do not bring too much satisfaction either, with mean value close to 25 %.

**Table 2:** Mean (arithmetic) chemical recoveries and their standard deviations for all analysed radionuclides

Element	Plutonium (%)	Americium (%)	Nickel (%)	Neptunium (%)	Strontium (%)	Thorium (%)	Uranium (%)
Mean recovery	51.3	43.6	7.8	34.7	62.4	61.3	24.2
Std. deviation	21.4	17.0	6.9	18.1	19.0	20.5	19.6

## Results and discussion

The results for gamma spectrometric measurements are presented in Table 3. The activity concentration of ^137^Cs are on a level of single Bq/kg (dry weight), ranging from 1.2 ± 0.8 to 7.1 ± 1.3 Bq/kg. In profiles P1, P6, P8 and P11 it seems to be distributed more or less evenly in all examined layers, what suggests, that soil was cultivated (ploughed) after the fallout. Only for profile P10 there is a systematic reduction of the radiocaesium activity concentration with depth. Obtained values in case of activity concentration of ^137^Cs are much lower than results received for northern Poland in our previous works. It ranged between 2.4 ± 0.3 and 437 ± 13 Bq/kg in soil profiles collected in Bory Tucholskie region [14] and from 6 ± 1 to 2346 ± 184 Bq/kg in forest litter coming from large area of north-eastern Poland [15].

**Table 3:** Activity concentration for ^137^Cs and ^40^K and calculated deposition of ^137^Cs found in all collected five profiles by means of high resolution, semiconductor gamma spectrometry. In sample code first number is coding location, second number of layer from top

Sample code	Layer thickness (cm)	Dry weight (g)	^137^Cs (Bq/kg)	^40^K (Bq/kg)
P1-1	3.5	483.25	6.4 ± 1.3	256 ± 33
P1-2	2	265.53	5.7 ± 1.0	444 ± 30
P1-3	2.5	304.97	3.0 ± 1.2	307 ± 28
P1-4	2.5	289.48	5.7 ± 1.3	288 ± 39
P1-5	2.5	268.67	5.6 ± 1.0	471 ± 27
P1-6	2.5	279.93	6.6 ± 1.1	267 ± 29
P1-7	2.5	278.81	3.8 ± 0.6	305 ± 20
P6-1	2.5	235.17	3.8 ± 1.4	331 ± 44
P6-2	3.5	438.89	5.5 ± 1.4	351 ± 30
P6-3	2.5	264.21	5.0 ± 1.2	335 ± 28
P6-4	2.5	268.83	4.2 ± 1.0	354 ± 26
P6-5	2.5	247.92	4.2 ± 0.8	337 ± 22
P6-6	2.5	279.75	4.2 ± 1.0	306 ± 24
P6-7	2.9	314.87	7.1 ± 1.3	315 ± 24
P6-8	2.5	252.66	4.5 ± 1.1	367 ± 28
P6-9	2.9	311.01	3.9 ± 0.9	340 ± 25
P8-1	3	305.89	7.2 ± 1.0	429 ± 29
P8-2	2	209.45	5.8 ± 1.2	358 ± 29
P8-3	2.5	223.62	6.3 ± 1.4	319 ± 37
P8-4	2	189.50	5.4 ± 1.2	356 ± 28
P8-5	3	312.10	4.3 ± 0.9	435 ± 30
P8-6	2.5	257.47	7.0 ± 1.4	306 ± 38
P8-7	3	336.81	4.2 ± 1.0	358 ± 28
P8-8	3.5	376.62	4.7 ± 1.2	337 ± 34
P8-9	3	329.98	4.8 ± 1.0	371 ± 27
P10-1	2.5	221.82	6.3 ± 0.4	443 ± 24
P10-2	3	274.95	5.9 ± 1.0	360 ± 30
P10-3	3.5	310.01	3.6 ± 1.1	378 ± 35
P10-4	3.5	313.98	1.2 ± 0.8	342 ± 33
P11-1	3.5	347.79	4.2 ± 1.0	234 ± 19
P11-2	2	211.29	2.7 ± 1.0	292 ± 25
P11-3	2.5	234.52	3.7 ± 1.1	188 ± 29
P11-4	2.5	294.46	3.8 ± 0.9	194 ± 22
P11-5	2.5	267.73	2.4 ± 0.5	123 ± 11
P11-6	3	303.18	4.3 ± 0.7	297 ± 19
P11-7	3	313.88	5.9 ± 1.0	224 ± 24

On the other hand, potassium (^40^K) activity concentration is not uniform, especially for profile P1, as one could expect for an arable soil mixed by ploughing. Perhaps this is a result of artificial fertilization which can happen from time to time. Profiles P6, P8, P10 are similar. In general, it should be noted that the activity concentration for ^40^K in investigated soil profiles are in line with the soil average content for the world, which is 400 Bq/kg [16]. Lower potassium content than average for the world and the lowest from all investigated profiles is observed only for profile P11—none of the results exceeds 300 Bq/kg.

Table 4 presents results of activity concentration for artificial radionuclides separated in radiochemical procedures followed by alpha spectrometry, liquid scintillation spectrometry and mass spectrometry. Activity concentrations of ^239+240^Pu range between 0.074 ± 0.009 Bq/kg and 0.392 ± 0.051 Bq/kg (dry weight) and do not show any clear tendency. Again our results are lower or much lower than those received in earlier works both for northern and southern Poland [14, 15, 17–20]. In soil samples from north part of Poland there were obtained following ranges for ^239+240^Pu: from 0.045 ± 0.005 to 0.730 ± 0.103 Bq/kg (Borne–Sulinowo region) [17] and from 0.03 ± 0.01 to 5.90 ± 0.30 Bq/kg (Bory Tucholskie) [14].

**Table 4:** Results obtained in course of sequential radiochemical procedure for ^238^Pu, ^239+240^Pu, ^63^Ni (all results below detection limits), ^241^Am, ^237^Np and, ^90^Sr for five profiles. Plutonium and americium determined by alpha spectrometry, nickel, strontium by liquid scintillation counting and Neptunium by mass spectrometry

Sample code	Layer thickness (cm)	^239+240^Pu (Bq/kg)	^238^Pu (Bq/kg)	^241^Am (Bq/kg)	^63^Ni (Bq/kg)	^237^Np (mBq/kg)	^90^Sr (Bq/kg)
P1a	5.5	0.125 ± 0.015	<0.04	0.12 ± 0.02	<12	<0.10	14.0 ± 0.9
P1b	5	0.175 ± 0.015	0.03 ± 0.01	0.09 ± 0.01	<17	<0.10	13.6 ± 0.9
P1c	7.5	0.209 ± 0.023	<0.04	0.06 ± 0.01	<14	0.096 ± 0.025	6.8 ± 0.5
P6a	6	0.259 ± 0.041	<0.02	0.31 ± 0.04	<21	<0.17	10.0 ± 0.6
P6b	7.5	0.095 ± 0.044	0.010 ± 0.003	0.12 ± 0.02	<12	0.47 ± 0.18	8.9 ± 0.6
P6c	11.5	0.167 ± 0.016	0.008 ± 0.004	0.13 ± 0.03	<36	0.367 ± 0.097	8.8 ± 0.5
P8a	7.5	0.222 ± 0.028	0.006 ± 0.002	0.02 ± 0.01	< 13	0.141 ± 0.037	12.9 ± 0.9
P8b	7.5	0.167 ± 0.025	0.028 ± 0.008	0.05 ± 0.02	<43	0.309 ± 0.084	10.8 ± 0.7
P8c	9.5	0.237 ± 0.026	<0.03	<0.04	<13	0.176 ± 0.045	12.0 ± 0.8
P10a	2.5	0.360 ± 0.034	0.024 ± 0.006	0.03 ± 0.01	<36	0.29 ± 0.28	11.1 ± 0.7
P10b	3	0.392 ± 0.051	0.068 ± 0.013	0.30 ± 0.05	<16	0.322 ± 0.104	11.9 ± 0.8
P10c	7	0.105 ± 0.021	<0.03	0.09 ± 0.01	<50	0.346 ± 0.094	9.8 ± 0.6
P11a	5.5	0.175 ± 0.018	0.037 ± 0.015	0.03 ± 0.01	<3	<0.12	8.5 ± 0.5
P11b	7.5	0.074 ± 0.009	<0.04	<0.10	<8	0.145 ± 0.037	12.1 ± 0.8
P11c	6	0.084 ± 0.018	<0.05	0.04 ± 0.01	<10	0.110 ± 0.029	11.9 ± 0.8

Received values of activity concentrations for ^238^Pu in soil from studied area are much lower than for ^239+240^Pu, indicating no significant influence of Chernobyl fallout. The minimum value is <0.02 Bq/kg, the maximum equals 0.068 ± 0.013 Bq/kg, while the maximum activity concentration of ^238^Pu in soil from Bory Tucholskie was higher and equelled 0.27 ± 0.03 Bq/kg [14]. On the other hand Komosa obtained lower maximum level in soil samples form Borne– Sulinowo region, that was 0.034 ± 0.004 Bq/kg [17].

The highest amount of ^238^Pu was observed in the middle part of P10 profile. However, there is not accompanied by a clear enhanced level of ^241^Am activity. Activity concentration of ^241^Am ranges from 0.02 ± 0.01 till up to 0.31 ± 0.04 Bq/kg. The last result (top 6 cm layer of P6 profile) is the only case when americium activity concentration is higher than plutonium one. The first two layers of profile P1 (down to 10.5 cm) are characterized by similar results for all artificial radionuclides, whereas for the third layer activity concentration of ^90^Sr is lower by a factor of two. Profile P6 shows a little higher Pu and Am content in first 6 cm and then in next 18 cm, while distribution of ^90^Sr seems to be rather uniform along the whole length of the profile.

In case of ^237^Np the minimum values of activity concentration is <0.1 Bq/kg and the maximum reaches 0.367 ± 0.097 Bq/kg. The range of our results for ^237^Np/^239+240^Pu activity ratio varied from 0.00046 ± 0.00013 to 0.0049 ± 0.0030 are wider than set of values presented in earlier works of Baesley et al. [21] for soil samples which ranged between 0.0024 ± 0.0001 and 0.0032 ± 0.0006. It confirms the results of pilot study for Poland and points to the global fallout as a predominant source of neptunium in the samples [10].

The summary of the total inventory (cumulated deposition) of artificial radionuclides is presented in Table 5.

**Table 5:** Total deposition (inventory) for artificial radionuclides in investigated five soil profiles

Site code	Total depth (cm)	^137^Cs (Bq/m^2^)	^90^Sr (Bq/m^2^)	^239+240^Pu (Bq/m^2^)	^238^Pu (Bq/m^2^)	^241^Am (Bq/m^2^)	^237^Np (mBq/m^2^)
P1	18	1474 ± 126	3082 ± 127	47.2 ± 3.1	2.27 ± 0.76	24.6 ± 2.3	10.1 ± 2.6
P6	24.3	1598 ± 132	3043 ± 114	56.3 ± 6.1	2.18 ± 0.66	57.7 ± 6.0	101 ± 23
P8	24.5	1747 ± 121	3854 ± 158	68.5 ± 5.0	3.27 ± 0.80	6.7 ± 2.2	67 ± 11
P10	12.5	575 ± 65	1509 ± 62	32.2 ± 2.7	3.06 ± 0.49	18.5 ± 2.0	47 ± 11
P11	19	997 ± 84	2769 ± 114	26.6 ± 2.1	2.6 ± 1.1	5.3 ± 1.1	23.4 ± 4.4

The inventory for ^137^Cs (determined for date of measurement) is lower than it would be expected based on the available data for Poland [22]. It ranges from 575 ± 65 (site P10, 12.5 cm depth of profile) to 1747 ± 121 Bq/m^2^ (site P8, 24.5 cm of total depth). Apparently not all profiles were taken deep enough to collect all deposited radiocaesium. It is similar for ^239+240^Pu. Profiles P10 and P11 contain about half of the mean expected value for latitude belt of Poland, which equals 58 Bq/m^2^ [23]. The comparison of the results for plutonium inventory and ^237^Np suggests some fractionation between Pu and Np. The ^237^Np ranges from 10.1 ± 2.5 (P1 profile) till up to 101 ± 23 mBq/m^2^ (profile P6). These results were comparable with previous obtained by Kelley et al. [24]. They found for soil samples from Northern Hemisphere values ranged between 50.8 ± 1.4 and 379 ± 13 mBq/m^2^ (data were calculated from the determined mass spectrometry atom results using following half life time of ^237^Np: 2,140,000 years).

Despite the relatively low levels of activity of each radioisotope, often affected by quite high uncertainty, the isotope ratios were calculated and the values are given in Table 6.

**Table 6:** Isotopic ratios (Summer 2014) for artificial radionuclides in five soil profiles

Sample code	^238^Pu/^239+240^Pu	^239+240^Pu/^137^Cs	^241^Am/^239+240^Pu	^239+240^Pu/^90^Sr	^137^Cs/^90^Sr
P1a		0.0204 ± 0.0033	0.96 ± 0.20	0.0089 ± 0.0012	0.438 ± 0.055
P1b	0.171 ± 0.059	0.0407 ± 0.0070	0.514 ± 0.074	0.0129 ± 0.0014	0.316 ± 0.052
P1c		0.0392 ± 0.0050	0.287 ± 0.058	0.0307 ± 0.0042	0.785 ± 0.076
P6a		0.053 ± 0.011	1.20 ± 0.25	0.0259 ± 0.0044	0.492 ± 0.078
P6b	0.105 ± 0.058	0.021 ± 0.010	1.26 ± 0.62	0.0107 ± 0.0050	0.500 ± 0.051
P6c	0.048 ± 0.024	0.0336 ± 0.0037	0.78 ± 0.20	0.0190 ± 0.0022	0.564 ± 0.044
P8a	0.027 ± 0.010	0.0340 ± 0.0048	0.090 ± 0.047	0.0172 ± 0.0025	0.506 ± 0.048
P8b	0.168 ± 0.054	0.0307 ± 0.0051	0.30 ± 0.13	0.0155 ± 0.0026	0.504 ± 0.049
P8c		0.0520 ± 0.0070		0.0198 ± 0.0026	0.380 ± 0.039
P10a	0.067 ± 0.018	0.0575 ± 0.0069	0.083 ± 0.029	0.0324 ± 0.0038	0.564 ± 0.054
P10b	0.173 ± 0.040	0.066 ± 0.014	0.77 ± 0.16	0.0329 ± 0.0049	0.498 ± 0.091
P10c		0.044 ± 0.012	0.86 ± 0.20	0.0107 ± 0.0023	0.244 ± 0.051
P11a	0.211 ± 0.089	0.0480 ± 0.0083	0.171 ± 0.060	0.0206 ± 0.0025	0.429 ± 0.064
P11b		0.0225 ± 0.0034		0.0061 ± 0.0009	0.272 ± 0.030
P11c		0.0164 ± 0.0038	0.48 ± 0.16	0.0071 ± 0.0016	0.431 ± 0.047

Table 7 summarizes the reference values of the isotopic ratios (corrected for decay) of the selected sources of radioactive contamination characteristic for territory covering Żarnowiec Lake area. Based on the analysis of the results and comparison them with reference values it can be seen again, that the global fallout is the dominant source of contamination in the examined soil profiles. Overview of the values of ^238^Pu/^239+240^Pu ratio indicates a possible slight impact of Chernobyl fallout in selected individual layers of each profile. However, these results are affected by relatively high uncertainties, so they can not constitute a basis for such statement.

**Table 7:** Isotopic ratios characteristic for chosen sources, region including study area and artificial radionuclides investigated in this paper

Type of source	^238^Pu/^239+240^Pu	^239+240^Pu/^137^Cs	^241^Am/^239+240^Pu	^239+240^Pu/^90^Sr	^137^Cs/^90^Sr
Global fallout + SNAP 9A	0.03^c^ [23]	0.023^b^ [23]	0.43^a^ [23]	0.04^c^ [23]	1.6^c^ [23, 27]
Chernobyl	0.24^c^ [28]–0.52^c^ [29]	0.000011^c, d^ [29]	2.7^b^ [25]	–	50^c, d^ [30]

^a^After complete decay of ^241^Pu in 2035 [23]

^b^After comlete decay of ^241^Pu in 2060 [25]

^c^Corrected for decay (2014)

^d^For remote from Chernobyl locations

Some kind of verification of the possible impact of Chernobyl fallout in investigated samples can be done by an analysis of ^241^Am/^239+240^Pu ratios. For the global fallout this ratio equals roughly 0.4, while for Chernobyl fallout is much higher, finally close to 3:1 [25]. In particular, elevated values of isotopic ratios were found in the upper layer of profile 1 and 6 and in the lower layers of the profile 10. Excess of ^241^Am might suggest fractionation between Pu and Am isotopes or (and) the presence of Chernobyl influence.

Additional information about sources of actinides in considered soil material were provided by results obtained by combined alpha and mass spectrometry. The assumption that mass ratio of ^240^Pu/^239^Pu for global fallout equalled 0.180 ± 0.036 allowed us to estimate ^237^Np/^239^Pu mass ratio. Our data (0.067 ± 0.018–0.72 ± 0.39) suit very well to the general global trend (0.028 ± 0.002–0.636 ± 0.028) establish by the worldwide research showed that average compositions of fallout Np and Pu isotopes in soil of Northern Hemisphere (latitude 70–30 N) is 0.48 ± 0.07 [21]. This conclusion confirmed that nuclear tests were the origin of artificial nuclides in soil from northern Poland.

Low impact of Chernobyl fallout of actinides presence on this area can be somehow unexpected, since in the studies conducted in 1990s in northern Poland [14, 17], even on the areas forwarded to the west, some fraction (even up to 15 %) of Pu from Chernobyl was determined. However, it was already found [2, 18], that the apparent percentage of Chernobyl origin plutonium is diminishing. Two explanations were proposed, perhaps both proper. In one study, Chernobyl-origin Pu was thought to be related with particles, which migrates faster down the soil profile then global-fallout Pu [18]. Another explanation [2] suggests that plutonium isotopic ratio is averaged within profile due to biological processes. Since Chernobyl fraction was important only in selected (top) layer, after its distribution through the whole profile its presence is much more difficult to observe.

Evaluation of ^137^Cs/^90^Sr ratios provided quite unexpected information. It can be seen that there is incomprehensible excess of ^90^Sr compared to ^137^Cs for each profile. Perhaps it is related to the specific properties of tested soil, namely more efficient sorption in soil profile of ^90^Sr compared to ^137^Cs. It can be also result of effective recycling of strontium in top soil (uptake by plants and then fall with decomposition of dead parts of plants) whereas caesium mostly migrates downward.

According to Table 8 there can be seen that received values of natural alfa emitters activity concentrations ranges from: 8.8 ± 0.7 to 19.2 ± 1.7 Bq/kg for ^232^Th, 7.2 ± 0.6 to 19.6 ± 1.3 Bq/kg for ^230^Th, 3.4 ± 0.3 to 18.5 ± 3.2 Bq/kg for ^234^U and from 3.8 ± 0.4 to 18.0 ± 2.4 Bq/kg in case of ^238^U. The uranium and thorium isotopes are present on the levels lower than one could expect in typical soil [26].

**Table 8:** Results obtained in course of sequential radiochemical procedure for natural isotopes ^234^U, ^238^U, ^232^Th, ^230^Th for combined samples of five profiles determined by alpha spectrometry

Sample	^232^Th (Bq/kg)	^230^Th (Bq/kg)	^234^U (Bq/kg)	^238^U (Bq/kg)
p1a	13.6 ± 1.0	13.6 ± 0.9	11.7 ± 2.5	14.0 ± 2.9
p1b	11.5 ± 1.0	11.7 ± 0.9	9.3 ± 1.7	14.0 ± 2.5
p1c	11.3 ± 0.9	12.5 ± 0.9	11.2 ± 1.5	11.5 ± 1.5
p6a	13.3 ± 1.0	15.9 ± 1.1	12.7 ± 1.9	15.3 ± 2.3
p6b	15.7 ± 1.1	16.8 ± 1.1	16.8 ± 2.4	17.8 ± 2.6
p6c	14.7 ± 1.2	16.2 ± 1.2	12.3 ± 1.5	12.8 ± 1.5
p8a	15.8 ± 1.3	17.2 ± 1.2	3.35 ± 0.3	3.8 ± 0.4
p8b	16.9 ± 1.2	16.9 ± 1.2	8.7 ± 1.4	13.2 ± 2.6
p8c	16.1 ± 1.1	17.1 ± 1.1	18.5 ± 3.2	17.1 ± 3.2
p10a	18.7 ± 1.3	19.6 ± 1.3	16.1 ± 1.8	18.0 ± 2.4
p10b	18.3 ± 1.5	17.8 ± 1.3	16.7 ± 2.9	16.9 ± 2.9
p10c	19.2 ± 1.7	19.3 ± 1.5	13.8 ± 1.8	15.2 ± 1.9

All results obtained for ^63^Ni are below the detection limit and ranged between <3 and <50 Bq/kg.

## Conclusions

The “time zero state” of radioactive contamination in Żarnowiec Lake area was determined by activity concentration or deposition calculated for examined radioisotopes is as follows:

(a) the total deposition obtained only for artificial radionuclides ranges: from 575 ± 65 to 1747 ± 121 Bq/m^2^ for ^137^Cs, from 1509 ± 62 to 3854 ± 158 Bq/m^2^ for ^90^Sr, from 10.1 ± 2.6 to 101 ± 23 mBq/m^2^ for ^237^Np, from 5.3 ± 1.1 to 57.7 ± 6.0 Bq/m^2^ for ^241^Am, in case of plutonium isotopes inventory varies between 2.18 ± 0.66 and 3.27 ± 0.80 Bq/m^2^ for ^238^Pu and from 26.6 ± 2.1 to 68.5 ± 5.0 Bq/m^2^ for ^239+240^Pu,

(b) for natural radionuclides activity concentrations range: from 123 ± 11 to 471 ± 27 Bq/kg for ^40^K, from 8.8 ± 0.7 to 19.2 ± 1.7 Bq/kg for ^232^Th, from 7.2 ± 0.6 to 19.6 ± 1.3 Bq/kg for ^230^Th, from 3.4 ± 0.3 to 18.5 ± 3.2 Bq/kg for ^234^U and from 3.8 ± 0.4 to 18.0 ± 2.4 Bq/kg in case of ^238^U.

Comparison of observed radioactive contamination with the data available for other Polish locations allows us to conclude, that investigated area doesn’t show any enhanced levels of either natural or artificial radionuclides. Studied area seems to be contaminated mostly by global fallout. No clear input of Chernobyl fallout was noticed, although the ratios between americium and plutonium inventories suggests some trace presence of Chernobyl origin americium and thus also plutonium.

Proposed sequential radiochemical procedure is reliable and works properly except for nickel-63, which needs some further work to enhance recoveries. For the first time upper limits for ^63^Ni in soil samples from Poland as well as results of ^237^Np inventory in a soil profiles are given.
